# Impact of pretreatment anemia on upfront abiraterone acetate therapy for metastatic hormone-sensitive prostate cancer: a multicenter retrospective study

**DOI:** 10.1186/s12885-021-08206-8

**Published:** 2021-05-25

**Authors:** Teppei Okamoto, Daisuke Noro, Shingo Hatakeyama, Shintaro Narita, Koji Mitsuzuka, Toshihiko Sakurai, Sadafumi Kawamura, Senji Hoshi, Jiro Shimoda, Toshikazu Tanaka, Toshiaki Kawaguchi, Shigeto Ishidoya, Akihiro Ito, Norihiko Tsuchiya, Tomonori Habuchi, Chikara Ohyama

**Affiliations:** 1 Department of Urology, Department of Advanced Blood Purification Therapy, Hirosaki University Graduate School of Medicine, 5 Zaifu-chou, Hirosaki, 036-8562 Japan; 2 Department of Urology, Akita University School of Medicine, 1-1-1, Hondo, Akita, 010-8543 Japan; 3 Department of Urology, Tohoku University School of Medicine, 2-1 Seiryo-machi, Aoba-ku, Sendai, Miyagi 980-8575 Japan; 4 Department of Urology, Yamagata University School of Medicine, 2-2-2 Iida-Nishi, Yamagata, Yamagata 990-9585 Japan; 5 Department of Urology, Miyagi Cancer Center, 47-1, Nodayama, Shiote, Aijima, Natori, Miyagi 981-1293 Japan; 6 Department of Urology, Yamagata Prefectural Central Hospital, 1800, Aoyanagi, Yamagata, 990-2292 Japan; 7 Department of Urology, Iwate Prefectural Isawa Hospital, 61, Ryugabaab, Mizusawa-ku, Oshu, Iwate, 023-0864 Japan; 8 Department of Urology, Aomori Prefectural Central Hospital, 2-1-1, Higashi-tsukurimichi, Aomori, Aomori 030-8553 Japan; 9 Department of Urology, Sendai City Hospital, 1-1-1, Nagamachi, Asuto, Taihaku-ku, Sendai, Miyagi 982-8502 Japan

**Keywords:** Prostate cancer, Hormone-sensitive, Abiraterone acetate, Upfront therapy, Prognosis, Anemia

## Abstract

**Background:**

Anemia has been a known prognostic factor in metastatic hormone-sensitive prostate cancer (mHSPC). We therefore examined the effect of anemia on the efficacy of upfront abiraterone acetate (ABI) in patients with mHSPC.

**Methods:**

We retrospectively evaluated 66 mHSPC patients with high tumor burden who received upfront ABI between 2018 and 2020 (upfront ABI group). We divided these patients into two groups: the anemia-ABI group (hemoglobin < 13.0 g/dL, *n* = 20) and the non-anemia-ABI group (*n* = 46). The primary objective was to examine the impact of anemia on the progression-free survival (PFS; clinical progression or PC death before development of castration resistant PC) of patients in the upfront ABI group. Secondary objectives included an evaluation of the prognostic significance of upfront ABI and a comparison with a historical cohort (131 mHSPC patients with high tumor burden who received androgen deprivation therapy (ADT/complete androgen blockade [CAB] group) between 2014 and 2019).

**Results:**

We found that the anemia-ABI group had a significantly shorter PFS than the non-anemia-ABI group. A multivariate Cox regression analysis showed that anemia was an independent prognostic factor of PFS in the upfront ABI group (hazard ratio, 4.66; *P* = 0.014). Patients in the non-anemia-ABI group were determined to have a significantly longer PFS than those in the non-anemia-ADT/CAB group (*n* = 68) (*P* < 0.001). However, no significant difference was observed in the PFS between patients in the anemia-ABI and the anemia-ADT/CAB groups (*n* = 63). Multivariate analyses showed that upfront ABI could significantly prolong the PFS of patients without anemia (hazard ratio, 0.17; *P* < 0.001), whereas ABI did not prolong the PFS of patients with anemia.

**Conclusion:**

Pretreatment anemia was a prognostic factor among mHSPC patients who received upfront ABI. Although the upfront ABI significantly improved the PFS of mHSPC patients without anemia, its efficacy in patients with anemia might be limited.

**Supplementary Information:**

The online version contains supplementary material available at 10.1186/s12885-021-08206-8.

## Background

Prostate cancer (PC) is one of the most prevalent cancers in men worldwide [1, 2]. In Japan, approximately 10% of patients with PC initially present with distant metastases [3]. Most of these patients experience eventually progression to metastatic castration-resistant PC (CRPC) despite good initial response to androgen deprivation therapy (ADT) [4, 5]. Recently, the LATITUDE trial demonstrated that upfront abiraterone acetate (ABI, a type of androgen receptor-targeted agent [ARTA]) added to ADT can lead to significant benefits in mHSPC patients compared with ADT monotherapy [6, 7]. However, these studies showed an almost similar PFS and overall survival between the ADT monotherapy and upfront ABI groups in the early term of the study [6, 7]. Thus, we speculated that some important prognostic and/or predictive factors may be present among patients who received ABI therapy.

Anemia is a powerful prognostic factor in PC [5, 8]. Our previous study demonstrated that pretreatment anemia was an independent prognostic factor that predicted oncological outcomes among mHSPC patients treated with ADT monotherapy or complete androgen blockade (CAB) [5]. However, the prognostic significance of anemia among mHSPC patients treated with ARTA remains unclear. Therefore, we retrospectively examined the prognostic significance of pretreatment anemia on the oncological outcomes of mHSPC patients treated with upfront ABI.

## Methods

This retrospective study was performed according to the ethical standards of the Declaration of Helsinki and was further approved by the ethics review board of the Hirosaki University School of Medicine (authorization number: 2019–094).

### Study population and patient selection

**Fig. 1:**
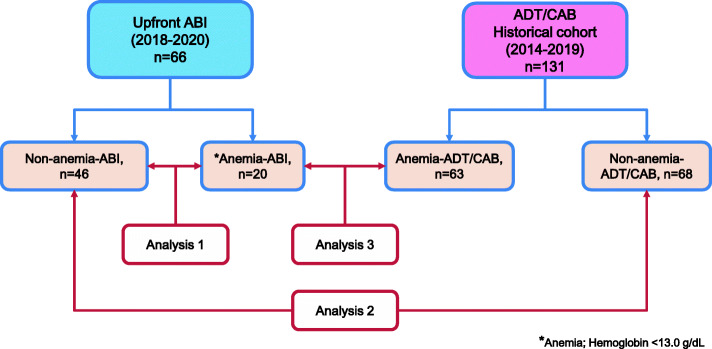
Patient evaluation. Patients with high tumor burden (CHAARTED high-volume and/or LATITUDE high-risk) were evaluated in this study. We identified 67 and 135 patients in the ADT/CAB and upfront groups, respectively. We divided the patients according to the presence of pretreatment anemia into the non-anemia-ABI, anemia-ABI, non-anemia-ADT/CAB, and anemia-ADT/CAB groups

In total, 168 mHSPC patients with high tumor burden, who were initially treated with ADT alone or CAB (ADT/CAB, *n* = 101) or upfront ABI therapy (*n* = 67), were retrospectively examined at the Hirosaki University Hospital and associated hospitals between 2008 and 2020 (Aomori database). Furthermore, we retrospectively evaluated 563 mHSPC patients with CHAARTED high-volume disease, who were in the Michinoku Japan Urological Cancer Study Group database and who were initially treated with ADT/CAB between 2008 and 2016 (Michinoku database) [4, 5, 9]. Considering that patients with metastatic CRPC had a chance to receive ARTAs and improved their prognosis, the era of diagnosis or treatment might be associated with prognoses among mHSPC patients. We therefore excluded 22 patients treated with ADT/CAB who were diagnosed before 2014 in the Aomori database and 131 in the Michinoku database. We excluded 22 patients treated with ADT/CAB in the Aomori database and 354 in the Michinoku database due to insufficient baseline laboratory data, such as information on hemoglobin (Hb), lactate dehydrogenase (LDH), and alkaline phosphatase (ALP) (Fig. S1). In addition, we excluded 5 patients (1 for upfront ABI group and 4 for ADT/CAB group) who died of other than PC before the development of CRPC. Finally, 197 mHSPC patients who had high tumor burden were evaluated (Fig. 1); they were then divided into two groups: the ADT/CAB group (*n* = 131) and the upfront ABI group (*n* = 66). We further categorized the patients into the following groups based on whether they had pretreatment anemia: non-anemia-ABI (*n* = 46), anemia-ABI (*n* = 20), non-anemia-ADT/CAB (*n* = 68), and anemia-ADT/CAB groups (*n* = 63).

### Variable evaluations

The following variables were examined at diagnosis: age, Eastern Cooperative Oncology Group Performance Status (ECOG-PS), Gleason score, and initial prostate-specific antigen (PSA), Hb, LDH, and ALP levels. ECOG-PS was defined as worse if ECOG-PS was of ≥1 [10]. The cutoff value of PSA level was set at 100 ng/mL [11]. Anemia was defined as an Hb level < 13.0 g/dL [12]. The cutoff values for LDH and ALP levels were set at 222 and 322 IU/L, respectively, based on the standard common reference for clinical laboratory tests in Japan [13]. We evaluated metastatic status before treatment using chest and body computed tomography and bone scintigraphy scans. Bone metastatic volume was assessed according to the extent of disease (EOD) score. EOD scores were divided into 5 based on bone scintigraphy as follows [14]. Score 0, normal or abnormal due to benign bone lesion; Score 1, less than 6 metastatic bone lesions, each of which is less than 50% the size of a vertebral body; Score 2, the number of metastatic bone lesions is between 6 and 20; Score 3, the number of metastatic bone lesions is more than 20 but less than a “super scan”; and Score 4, “super scan”. CRPC-free survival was evaluated from the date of the initial diagnosis of mHSPC to the date of CRPC diagnosis according to the recommendations of the Cancer Clinical Trials Working Group 2 [15].

### Treatment protocol

Patients were initially treated with ADT alone or ADT plus bicalutamide before upfront ABI therapy was approved. Starting in March 2018, patients who met the LATITUDE study criteria could receive upfront ABI therapy.

### Primary objective

Our primary purpose was to evaluate cancer progression-free survival (PFS) (i.e., development of CRPC or PC death before the development of CRPC). We compared the clinical PFS between patients in the anemia-ABI and those in the non-anemia-ABI groups (**Analysis-1**). The adverse event (AE)-related discontinuation of ABI was compared between patients with and without anemia. We then assessed the impact of anemia and other clinical factors on the PFS of patients in the upfront ABI group using Cox proportional hazard analyses. We adopted the propensity score-based inverse probability of treatment weighting (IPTW) method in order to adjust for group imbalances.

### Secondary objectives

The secondary objectives of this study were to compare the PFS between patients in the non-anemia-ABI group and those in the non-anemia-ADT/CAB group (**Analysis-2**), and between patients in the anemia-ABI group and those in the anemia-ADT/CAB group (**Analysis-3)**. We then assessed the efficacy of upfront ABI on the PFS using multivariate Cox proportional hazard analyses or IPTW-adjusted analyses.

### Statistical analysis

Statistical analyses were conducted using GraphPad Prism 5.03 (GraphPad Software, San Diego, CA, USA), EzR (R commander version 1.6–3), and R 3.3.2 (The R Foundation for Statistical Computing, Vienna, Austria). Categorical variables were compared using Fisher’s exact test or chi-squared test. Quantitative variables are then expressed as means with standard deviations or as medians with interquartile ranges (IQRs). The statistical difference between groups was compared using Student’s *t*-test for normally distributed data or Mann–Whitney *U*-test for non-normally distributed data. The PFS was estimated and compared using the Kaplan–Meier curve and log-rank test, respectively. In case of limited event numbers, we adopted an IPTW-adjusted multivariable Cox regression analysis. Hazard ratios (HRs) with 95% confidence intervals (CIs) were calculated after controlling for potential confounders of PC, such as age, ECOG-PS, initial PSA, Gleason score, visceral metastasis, EOD, and ALP and LDH levels. We also conducted a simultaneous multivariate Cox regression analysis of PFS. *P*-values < 0.05 were considered to be statistically significant.

## Results

### Primary objective

**Fig. 2:**
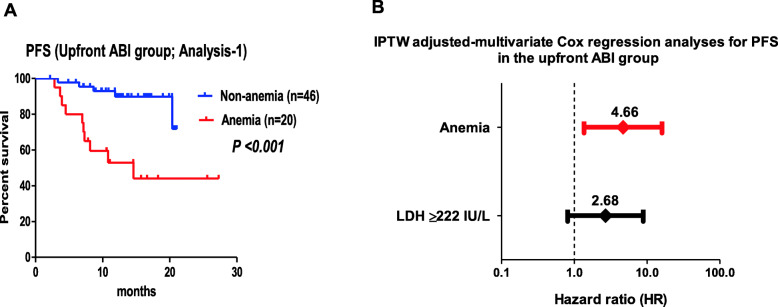
Comparison of clinical progression (Analysis-1). **a** PFS between patients in the anemia-ABI and non-anemia-ABI groups (Analysis-1) (median, not reached vs. 15 months; *P* < 0.001). **b** IPTW adjusted-multivariate logistic regression analyses for PFS in the upfront ABI group (adjustments for age, ECOG-PS, initial PSA, Gleason score, visceral metastasis, EOD, and ALP and LDH/Hb levels). Anemia (HR, 4.66; 95% CI, 1.36–16.0 *P* = 0.014). LDH level > 222 IU/L (HR, 2.68; 95% CI, 0.81–8.82 *P* = 0.105)

The patients in the anemia-ABI had significantly a worse ECOG-PS (*P* = 0.039), higher initial PSA (*P* = 0.001), EOD (*P* = 0.002), ALP (*P* = 0.010), and LDH (*P* = 0.017) than those in the non-anemia-ABI group (Table 1). Three patients discontinued ABI therapy due to severe AEs (two for liver damage and one for hyponatremia). No difference was observed in the rate of AE-related discontinuation of ABI between the two groups. During the median follow-up period of 13 months, 15 patients in the upfront ABI group experienced clinical progression (Table 1). Fourteen patients developed CRPC (nine in the anemia-ABI group), and two patients in the anemia-ABI group died before the development of CRPC. Of the 14 patients who developed CRPC, 2 died from PC. We observed a significantly longer PFS in the non-anemia-ABI group compared with that of the anemia-ABI group (*P* < 0.001) **(**Fig. 2a). Univariate Cox regression analyses in the ABI group showed that an LDH level ≥ 222 IU/L (HR, 3.70; *P* = 0.019) and anemia (HR, 5.58; *P* = 0.002) were significantly associated with worse PFS (Table 2). IPTW-adjusted multivariate Cox regression analyses revealed that anemia (HR, 4.66; *P* = 0.014) was independently associated with worse PFS, while an LDH level ≥ 222 IU/L was not (Fig. 2b). A simultaneous multivariate Cox regression analysis of PFS showed that only anemia was an independent factor (HR, 5.64; *P* = 0.016; Table 3).

**Table 1:** Upfront ABI group

	Anemia	Non-anemia	*P* value
n	20	46	
Age^a^ (years) (mean, SD)	72 (6)	71 (6)	0.231
ECOG-PS^b^ (median, IQR)	0 (0-1)	0 (0-0)	0.039
Initial PSA^b^ (ng/mL) (median, IQR)	961 (314-2702)	189 (51-448)	0.001
Gleason score^b^ (median, IQR)	9 (8-10)	9 (8-9)	0.649
Visceral metastasis^c^	3 (15%)	11 (24%)	0.524
Lung	2	11	-
Liver	1	0	-
Others	0	0	-
EOD^b^ (median, IQR)	3 (2-3)	2 (2-3)	0.002
ALP^b^ (IU/L) (median, IQR)	949 (372-1684)	357 (258-717)	0.010
LDH^b^ (IU/L) (median, IQR)	233 (200-279)	199 (166-227)	0.017
Discontinuation of ABI due to AEs^c^	1 (5.0%)	2 (4.3%)	1.000
Clinical progression^c^ (CRPC or PC death before CRPC)	10 (50%)	5 (11%)	<0.001
CRPC^c^	9 (43%)	5 (11%)	0.002
PC death before CRPC^c^	1 (5.0%)	0 (0%)	0.303
Death after CRPC^c^	1 (5.0%)	1 (2.3%)	0.518
Follow-up period^b^ (months) (median, IQR)	11 (11-15)	13 (11-17)	0.130

*ABI* abiraterone acetate, *SD* standard deviation, *ECOG-PS* Eastern Cooperative Oncology Group performance status, *IQR* interquartile range, *PSA* prostate specific antigen, *EOD* extent of disease, *ALP* alkaline phosphatase, *LDH* lactate dehydrogenase, *AE* adverse event, *PC* prostate cancer, *CRPC* castration resistant prostate cancer

^a^Student t test

^b^Mann–Whitney U test

^c^ χ2 test or Fisher’s exact test

**Table 2:** Univariate Cox hazard proportional analyses for progression-free survival in the upfront ABI group

Variables	Factor	*P* value	Hazard ratio	95% CI
Age	≥75 years	0.780	0.85	0.27-2.78
ECOG-PS	≥1	0.150	2.09	0.76-5.75
Initial PSA	≥100 ng/ml	0.230	2.60	0.54-12.5
Gleason score	≥9	0.438	0.67	0.24-1.85
Visceral metastasis	presence	0.380	0.51	0.11-2.29
EOD	0-4	0.375	1.29	0.74-2.24
ALP	≥322 IU/L	0.290	1.97	0.56-6.98
LDH	≥222 IU/L	0.019	3.70	1.24-11.0
Anemia	presence	0.002	5.58	1.90-16.4

*ABI* abiraterone acetate, *CI* confidence interval, *ECOG-PS* Eastern Cooperative Oncology Group performance status, *PSA* prostate specific antigen, *EOD* extent of disease, *ALP* alkaline phosphatase, *LDH* lactate dehydrogenase

**Table 3:** Simultaneous multivariate Cox regression analysis for PFS in the upfront ABI group

Variable	Factor	*P* value	Hazard ratio	95% CI
Age ≥75 years	Presence	0.280	0.51	0.15-1.73
ECOG-PS ≥1	Presence	0.750	1.15	0.35-3.81
Initial PSA ≥100 ng/ml	Presence	0.810	0.92	0.14-5.96
Gleason score ≥9	Presence	0.910	0.94	0.31-2.82
Visceral metastasis	Presence	0.760	0.67	0.13-3.46
EOD	0-4	0.380	0.74	0.38-1.44
ALP ≥322 IU/L	Presence	0.940	0.94	0.19-4.59
LDH ≥222 IU/L	Presence	0.055	3.51	0.97-12.3
Anemia	Presence	0.016	5.64	1.39-20.9

*PFS* progression-free survival, *ABI* abiraterone acetate, *CI* confidence interval, *ECOG-PS* Eastern Cooperative Oncology Group performance status, *PSA* prostate specific antigen, *EOD* extent of disease, *ALP* alkaline phosphatase, *LDH* lactate dehydrogenase

### Secondary objectives

**Fig. 3:**
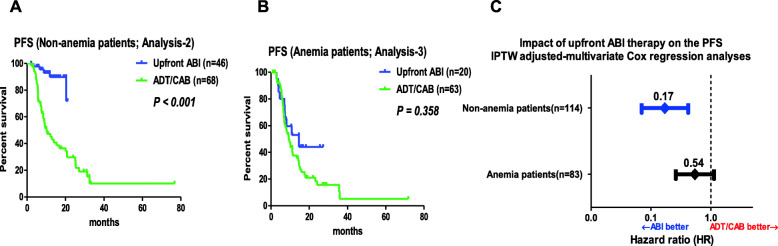
Comparison of clinical progression (Analysis-2, 3). **a** PFS between patients in the non-anemia-ABI and non-anemia-ADT/CAB groups (Analysis-2) (median, not reached vs. 10.1 months; *P* < 0.001). **b** PFS between patients in the anemia-ABI and anemia-AD/CAB groups (Analysis-3) (median, 15 vs. 9 months; *P* = 0.358). **c** IPTW adjusted-multivariate Cox regression analyses for PFS of upfront ABI (adjustment for age, ECOG PS, initial PSA, Gleason score, visceral metastasis, EOD, and ALP and LDH levels). Top for the non-anemia patients; (HR, 0.17; 95% CI, 0.07–0.42; *P* < 0.001). Bottom for the anemia patients; (HR, 0.54; 95% CI, 0.31–1.22; *P* = 0.186)

Table S2 illustrates a comparison of the characteristics between patients in the upfront ABI and ADT/CAB groups, according to the presence or absence of anemia. No significant group-difference was observed other than follow-up period between the non-anemia-ABI and non-anemia-ADT/CAB group, between the anemia-ABI and anemia-ADT/CAB group. Our additional study showed that patients in the upfront ABI group had a significantly longer PFS than those in the ADT/CAB group in the entire cohort (*P* < 0.001) (Fig. S2A). Similarly, the PFS in the non-anemia-ABI group was significantly superior to that in the non-anemia-ADT/CAB group (*P* < 0.001) (Fig. 3a). However, the PFS of patients in the anemia-ABI group did not have a significantly longer than that of patients in the anemia-ADT/CAB group (*P* = 0.358) (Fig. 3b). The IPTW adjusted-multivariable Cox regression analysis revealed that upfront ABI therapy was significantly associated with prolonged PFS in patients without anemia (HR, 0.17; *P* < 0.001) (Fig. 3c **top**), whereas this was not observed in patients with anemia (HR, 0.54; *P* = 0.186) (Fig. 3c **bottom**).

## Discussion

We compared the PFS between mHSPC patients with anemia and those without anemia in the upfront ABI group. We found an independent association between pretreatment anemia and the efficacy of upfront ABI therapy. As the LATITUDE trial has shown [6, 7], our study demonstrated the efficacy of upfront ABI therapy for mHSPC patients with high-tumor burden. This trend was remarkable especially in patients without anemia. However, we did not observe any meaningful difference in PFS between patients with anemia in the upfront ABI group and those in the ADT/CAB group. To the best of our knowledge, this is the first study demonstrating between pretreatment anemia and clinical outcome in patients treated with ARTA.

Anemia has been identified as one of the most prevalent characteristics of malignancies [16]. Among mHSPC patients, the prevalence of pretreatment anemia was as high as 44–50% [5, 17]. This study showed that the prevalence of anemia in this entire cohort was 43%. Anemia in mHSPC patients may be attributed to several factors, including malnutrition and chronic inflammation [5, 18]. Our previous study demonstrated that 85% of mHSPC patients with malnutrition were diagnosed with anemia [5]. Furthermore, the Hb level was inversely correlated with interleukin-6 expression, oxidative stress markers, and C-reactive protein level among patients with advanced cancer, which implied that inflammatory status is associated with worse iron metabolism in advanced cancers [16]. The relationship among anemia, malnutrition, and inflammatory status may be largely explained by cancer cachexia. Cancer cachexia is a complex syndrome that is often characterized by severe fatigue and progressive weight loss and is caused by a cancer-induced negative protein and energy balance as well as by inflammation. Malnutrition and inflammation suppress protein synthesis, particularly in advance caners, and it may also suppress erythropoiesis [19]. Another possible explanation for anemia in mHSPC patients is bone marrow infiltration. In this study, patients in the anemia-ABI group had a significantly worse EOD than those in the non-anemia-ABI group. Additionally, our study demonstrated that EOD was an independent factor of pretreatment anemia (Odds ratio, 1.93) in the entire cohort (Fig. S2B). Anemia due to replacement of normal bone marrow with cancer cells is termed leucoerythroblastic anemia [18], which is observed in approximately 30% of patients with metastatic CRPC [20]. These findings suggest that anemia among advanced PC patients may reflect cancer cachexia and/or disease aggressiveness.

The relationship between anemia and prognosis in patients with metastatic PC is debatable. A meta-analysis demonstrated that of the mHSPC patients in the ADT/CAB group, those with anemia had a significantly shorter PFS than those without anemia [8]. Tumor hypoxia caused by anemia and ADT may explain the relationship between anemia and poor prognosis in metastatic PC. Cancer-associated systemic anemia can cause decreased oxygen transport capacity of the blood, which contributes to hypoxia in the tumor microenvironment [21, 22]. Hypoxia may decrease tumor control through the induction of hypoxia-inducible factor 1α (HIF-1α) [23]. HIF-1α has been identified to activate HIF-1β to form a transcription factor complex that regulates the expression of several genes, such as vascular endothelial growth factor and glucose transporters, which is strongly associated with angiogenesis and tumor growth [24, 25]. Tumor hypoxia is often associated with treatment resistance in other cancers [23]. Tumor hypoxia in PC cell was associated with clinical stage and biochemical recurrence in locally advanced prostate cancer [26, 27]. Furthermore, ADT may accelerate hypoxia in PC cells, which ultimately results in CRPC [28]. Although the relationship between tumor hypoxia and responsiveness to ADT in mHSPC remains unclear, we speculated that poor clinical outcomes of mHSPC patients with anemia might be related with tumor hypoxia. Therefore, improvement in the prognosis of mHSPC patients with anemia is a key issue for clinicians.

Optimal selection of upfront intensive therapies for mHSPC patients with anemia must be carefully considered. Patients with anemia and/or malnutrition (cancer cachexia) are believed to be intolerant to chemotherapies [29]. In this regard, patients with anemia and/or malnutrition should be treated with upfront ABI therapy rather than upfront docetaxel. Indeed, the rate of AE-related discontinuation of ABI among patients with anemia was not very high in this study. However, our results implied that the efficacy of upfront ABI therapy might be limited in mHSPC patients with anemia. As no clinical trial of ARTAs focused on the impact of anemia on prognosis, further studies are necessary to confirm our findings. Careful selection for upfront ABI therapy might be necessary in mHSPC patients with anemia.

This study included a small sample size and a short-term follow-up period, which precluded a definitive conclusion regarding the long-term survival benefit. We did not obtain a full dataset on nutritional status and other immeasurable confounding factors. In this study, we therefore had to exclude 376 patients who received ADT/CAB due to lacking essential data, which resulted in major selection bias. We did not have data on baseline serum testosterone levels which is one of important factor of prostate cancer and anemia. Our results may not be applicable to other countries because of racial and regional differences. Despite these limitations, we revealed the prognostic significance of pretreatment anemia among mHSPC patients who received upfront ABI therapy.

## Conclusion

The upfront ABI therapy significantly improved the PFS of mHSPC patients, especially those without anemia. However, its efficacy in patients with anemia might be limited. Studies with a larger sample size and longer follow-up are needed to confirm our results.

## Supplementary Information

**Additional file 1: Figure S1.** Patient selection. We retrospectively evaluated 168 mHSPC patients with high tumor burden who were initially treated with ADT alone or CAB (ADT/CAB, *n* = 101) or upfront ABI therapy (*n* = 67) in the Aomori database. Furthermore, we retrospectively evaluated 563 mHSPC patients with high-volume disease, as reported in the CHAARTED trial, in the Michinoku database. We excluded 44 patients who were treated with ADT/CAB in the Aomori database and 485 patients in the Michinoku database due to old era of initial diagnosis (22 patients in the Aomori database and 131 in the Michinoku database) or insufficient baseline laboratory data or (22 patients in the Aomori database and 354 in the Michinoku database). **Figure S2** Comparison of clinical progression in the entire cohort and multivariate logistic analysis for pretreatment anemia. (**A**) PFS between patients in the upfront ABI and ADT/CAB groups and those in the entire cohort (median, not reached vs. 10 months; *P* < 0.001). (**B**) EOD (odds ratio [OR], 1.93; 95% CI, 1.27–1.93; *P* = 0.002) and age ≥ 75 years (OR, 3.16; 95% CI, 1.64–6.10; *P* < 0.001) were independent factors for pretreatment anemia. The model was adjusted by ALP ≥322 IU/L, LDH ≥222 IU/L, ECOG-PS ≥1, visceral metastasis, Gleason score ≥ 9, and initial PSA ≥100 ng/ml. Variance of inflation factor values of age ≥ 75 years, ALP ≥322 IU/L, EOD, LDH ≥222 IU/L, ECOG-PS ≥1, visceral metastasis, Gleason score ≥ 9, and initial PSA ≥100 ng/ml were 1.07, 1.15. 1.30, 1.05, 1.12, 1.17, 1.06, and 1.24, respectively, which means no multi-collinearity among covariates were observed.

## Data Availability

The datasets during and/or analyzed during the current study available from the corresponding author on reasonable request

## Abbreviations

mHSPC: metastatic hormone-sensitive prostate cancer
ABI: Abiraterone acetate
PFS: Progression-free survival
ARTA: Androgen receptor-targeted agent
ADT: Androgen deprivation therapy
CAB: Complete androgen blockage
PC: Prostate cancer
CRPC: Castration resistant prostate cancer
Hb: Hemoglobin
LDH: Lactate dehydrogenase
ALP: Alkaline phosphatase
ECOG-PS: Eastern Cooperative Oncology Group Performance Status
PSA: Prostate specific antigen
EOD: Extent of disease
AE: Adverse event
IPTW: Inverse probability of treatment weighting
HR: Hazard ratio
CI: Confidence interval
HIF: Hypoxia-inducible factor
