# Supplementary material for: Impact of pretreatment anemia on upfront abiraterone acetate therapy for metastatic hormone-sensitive prostate cancer: a multicenter retrospective study
Source: BMC Cancer. 2021 May 25;21:605. doi: 10.1186/s12885-021-08206-8 (PMC8152305; doi:10.1186/s12885-021-08206-8)
Supplement: Supplementary file 1 — Additional file 1: Figure S1. Patient selection. We retrospectively evaluated 168 mHSPC patients with high tumor burden who were initially treated with ADT alone or CAB (ADT/CAB, n = 101) or upfront ABI therapy (n = 67) in the Aomori database. Furthermore, we retrospectively evaluated 563 mHSPC patients with high-volume disease, as reported in the CHAARTED trial, in the Michinoku database. We excluded 44 patients who were treated with ADT/CAB in the Aomori database and 485 patients in the Michinoku database due to old era of initial diagnosis (22 patients in the Aomori database and 131 in the Michinoku database) or insufficient baseline laboratory data or (22 patients in the Aomori database and 354 in the Michinoku database). Figure S2 Comparison of clinical progression in the entire cohort and multivariate logistic analysis for pretreatment anemia. (A) PFS between patients in the upfront ABI and ADT/CAB groups and those in the entire cohort (median, not reached vs. 10 months; P < 0.001). (B) EOD (odds ratio [OR], 1.93; 95% CI, 1.27–1.93; P = 0.002) and age ≥ 75 years (OR, 3.16; 95% CI, 1.64–6.10; P < 0.001) were independent factors for pretreatment anemia. The model was adjusted by ALP ≥322 IU/L, LDH ≥222 IU/L, ECOG-PS ≥1, visceral metastasis, Gleason score ≥ 9, and initial PSA ≥100 ng/ml. Variance of inflation factor values of age ≥ 75 years, ALP ≥322 IU/L, EOD, LDH ≥222 IU/L, ECOG-PS ≥1, visceral metastasis, Gleason score ≥ 9, and initial PSA ≥100 ng/ml were 1.07, 1.15. 1.30, 1.05, 1.12, 1.17, 1.06, and 1.24, respectively, which means no multi-collinearity among covariates were observed. [file 12885_2021_8206_MOESM1_ESM.pptx]

## Slide 1
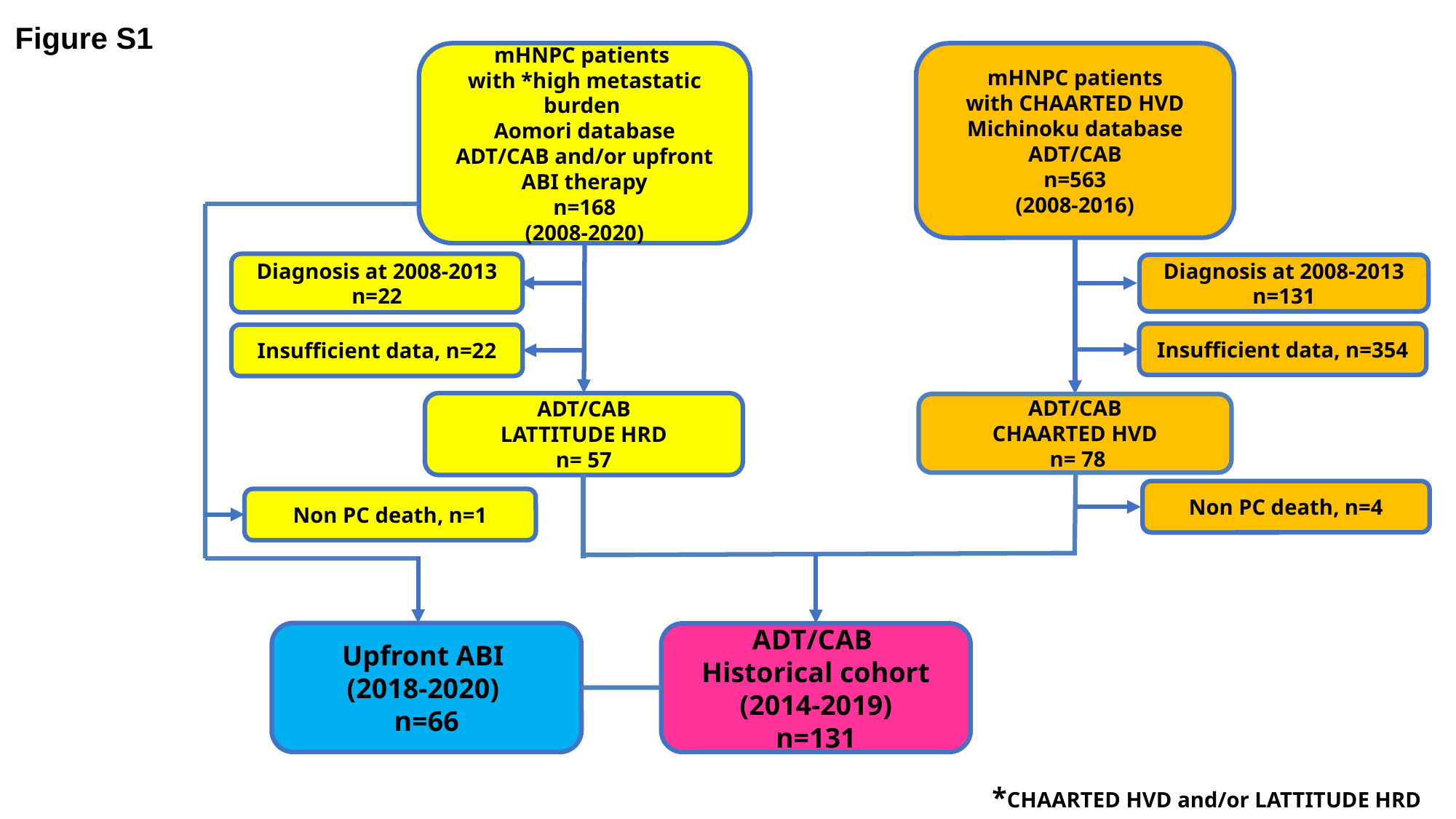

Figure S1
mHNPC patients
with *high metastatic burden
Aomori database
ADT/CAB and/or upfront ABI therapy
n=168
(2008-2020)
mHNPC patients
with CHAARTED HVD
Michinoku database
ADT/CAB
n=563
(2008-2016)
Diagnosis at 2008-2013
n=22
Diagnosis at 2008-2013
n=131
Insufficient data, n=354
Insufficient data, n=22
ADT/CAB
LATTITUDE HRD
n= 57
ADT/CAB
CHAARTED HVD
 n= 78
Non PC death, n=4
Non PC death, n=1
Upfront ABI
(2018-2020)
n=66
ADT/CAB
Historical cohort
(2014-2019)
n=131
*CHAARTED HVD and/or LATTITUDE HRD

## Slide 2
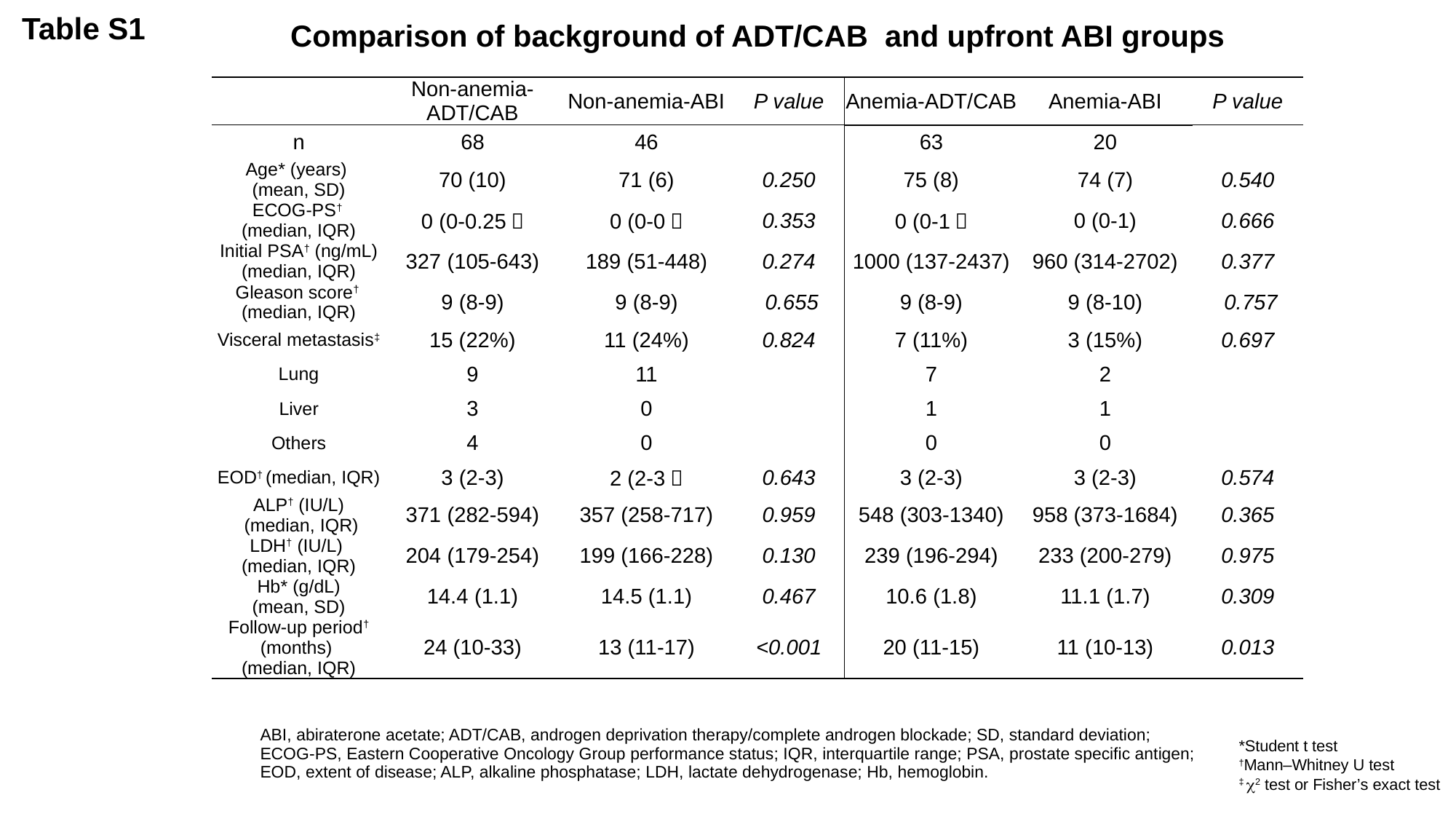

Table S1
Comparison of background of ADT/CAB and upfront ABI groups
| | Non-anemia-ADT/CAB | Non-anemia-ABI | P value | Anemia-ADT/CAB | Anemia-ABI | P value |
| --- | --- | --- | --- | --- | --- | --- |
| n | 68 | 46 | | 63 | 20 | |
| Age\* (years) (mean, SD) | 70 (10) | 71 (6) | 0.250 | 75 (8) | 74 (7) | 0.540 |
| ECOG-PS† (median, IQR) | 0 (0-0.25） | 0 (0-0） | 0.353 | 0 (0-1） | 0 (0-1) | 0.666 |
| Initial PSA† (ng/mL) (median, IQR) | 327 (105-643) | 189 (51-448) | 0.274 | 1000 (137-2437) | 960 (314-2702) | 0.377 |
| Gleason score† (median, IQR) | 9 (8-9) | 9 (8-9) | 0.655 | 9 (8-9) | 9 (8-10) | 0.757 |
| Visceral metastasis‡ | 15 (22%) | 11 (24%) | 0.824 | 7 (11%) | 3 (15%) | 0.697 |
| Lung | 9 | 11 | | 7 | 2 | |
| Liver | 3 | 0 | | 1 | 1 | |
| Others | 4 | 0 | | 0 | 0 | |
| EOD† (median, IQR) | 3 (2-3) | 2 (2-3） | 0.643 | 3 (2-3) | 3 (2-3) | 0.574 |
| ALP† (IU/L) (median, IQR) | 371 (282-594) | 357 (258-717) | 0.959 | 548 (303-1340) | 958 (373-1684) | 0.365 |
| LDH† (IU/L) (median, IQR) | 204 (179-254) | 199 (166-228) | 0.130 | 239 (196-294) | 233 (200-279) | 0.975 |
| Hb\* (g/dL) (mean, SD) | 14.4 (1.1) | 14.5 (1.1) | 0.467 | 10.6 (1.8) | 11.1 (1.7) | 0.309 |
| Follow-up period† (months) (median, IQR) | 24 (10-33) | 13 (11-17) | <0.001 | 20 (11-15) | 11 (10-13) | 0.013 |
| ABI, abiraterone acetate; ADT/CAB, androgen deprivation therapy/complete androgen blockade; SD, standard deviation; ECOG-PS, Eastern Cooperative Oncology Group performance status; IQR, interquartile range; PSA, prostate specific antigen; EOD, extent of disease; ALP, alkaline phosphatase; LDH, lactate dehydrogenase; Hb, hemoglobin. |
| --- |
*Student t test
†Mann–Whitney U test
‡ χ2 test or Fisher’s exact test

## Slide 3
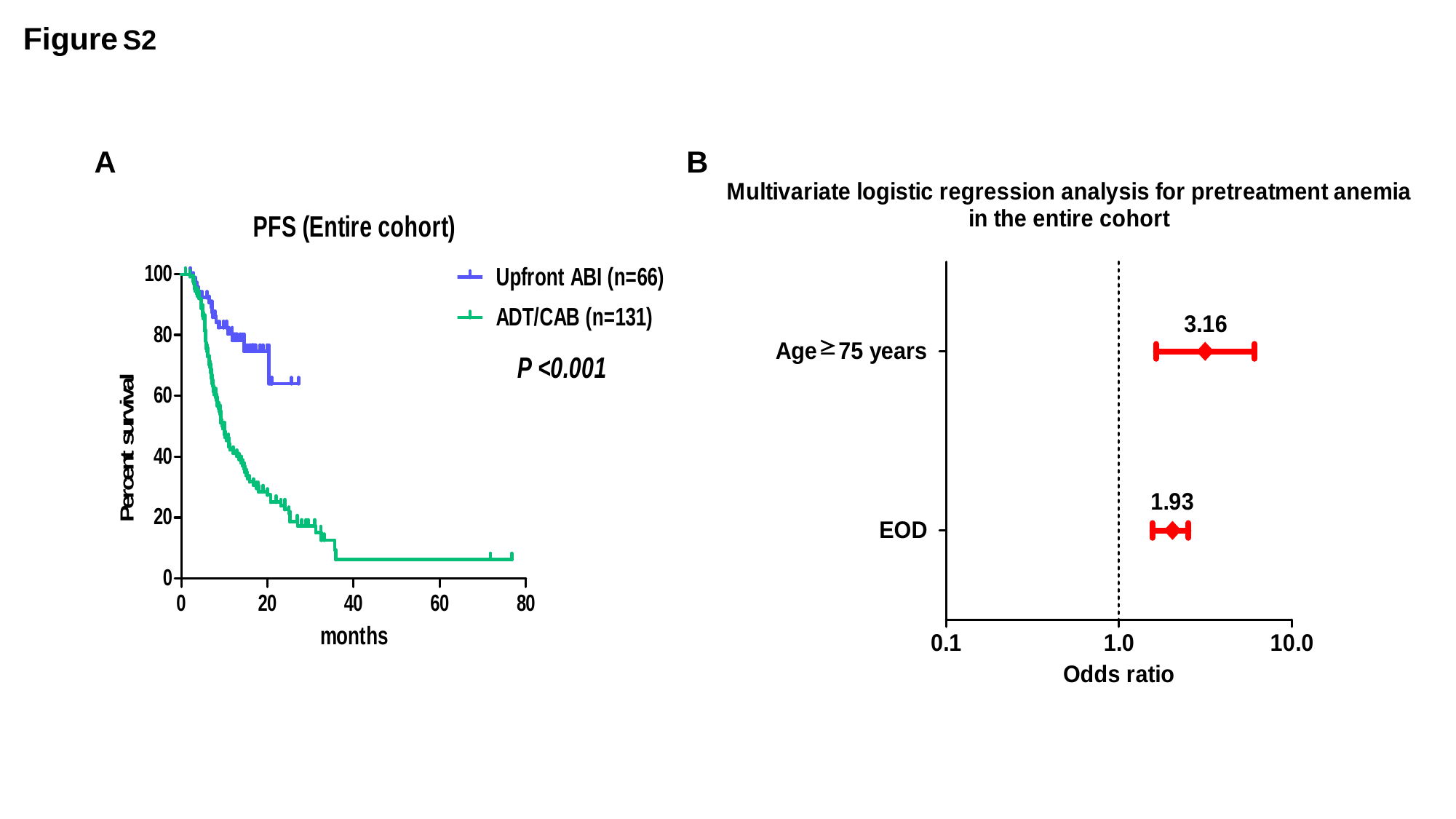

Figure S2
A
B
